# Structural Context of a Critical Exon of Spinal Muscular Atrophy Gene

**DOI:** 10.3389/fmolb.2022.928581

**Published:** 2022-07-01

**Authors:** Natalia N. Singh, Collin A. O'Leary, Taylor Eich, Walter N. Moss, Ravindra N. Singh

**Affiliations:** ^1^ Department of Biomedical Science, Iowa State University, Ames, IA, United States; ^2^ Roy J. Carver Department of Biochemistry, Biophysics and Molecular Biology, Iowa State University, Ames, IA, United States

**Keywords:** RNA structure, splicing, small molecule, spinal muscular atrophy, SMA, survival motor neuron, SMN, ISS-N1

## Abstract

Humans contain two nearly identical copies of *Survival Motor Neuron* genes, *SMN1* and *SMN2*. Deletion or mutation of *SMN1* causes spinal muscular atrophy (SMA), one of the leading genetic diseases associated with infant mortality. *SMN2* is unable to compensate for the loss of *SMN1* due to predominant exon 7 skipping, leading to the production of a truncated protein. Antisense oligonucleotide and small molecule-based strategies aimed at the restoration of *SMN2* exon 7 inclusion are approved therapies of SMA. Many cis-elements and transacting factors have been implicated in regulation of *SMN* exon 7 splicing. Also, several structural elements, including those formed by a long-distance interaction, have been implicated in the modulation of *SMN* exon 7 splicing. Several of these structures have been confirmed by enzymatic and chemical structure-probing methods. Additional structures formed by inter-intronic interactions have been predicted by computational algorithms. *SMN* genes generate a vast repertoire of circular RNAs through inter-intronic secondary structures formed by inverted Alu repeats present in large number in *SMN* genes. Here, we review the structural context of the exonic and intronic cis-elements that promote or prevent exon 7 recognition. We discuss how structural rearrangements triggered by single nucleotide substitutions could bring drastic changes in *SMN2* exon 7 splicing. We also propose potential mechanisms by which inter-intronic structures might impact the splicing outcomes.

## Introduction

**FIGURE 1:**
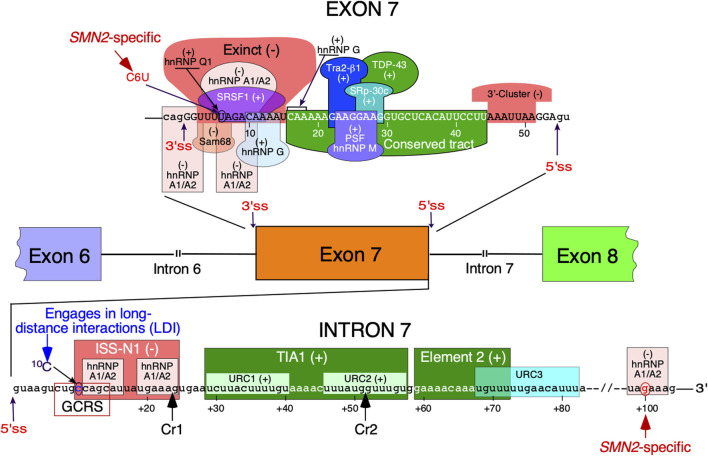
Regulation of *SMN* exon 7 splicing. Diagrammatic representation of intronic and exonic cis-elements as well as trans-acting factors that modulate *SMN* exon 7 splicing. Upper-case letters signify exonic sequences, small-case letters, intronic sequences. Exons and introns are also shown as colored boxes and lines, respectively. Numbering of nucleotides, neutral and positive, starts from the first exonic and intronic position, respectively. The 5 and 3′ splice sites (5′ss and 3′ss) are indicated by the arrows. Exinct, conserved tract and the 3′-Cluster are cis-elements revealed by *in vivo* selection as described in (Singh et al., 2004b). Cr1 and Cr2 represent cryptic 5′ splice sites as described in (Singh et al., 2017a). Negative and positive regulators of exon 7 splicing are indicated by (−) and (+), respectively. Abbreviations: Exinct, extended inhibitory context, GCRS, GC-rich region; LDI, long-distance interaction; URC, Uridine-rich clusters.

Survival Motor Neuron (SMN) protein is an essential housekeeping protein involved in multiple processes, including DNA replication and repair, transcription, pre-mRNA splicing, translation, macromolecular trafficking, stress granule formation, cell cycle regulation, signal transduction and maintenance of cytoskeletal dynamics (Singh R. N. et al., 2017). Low levels of SMN due to deletion or mutation of *SMN1* gene causes spinal muscular atrophy (SMA), one of the leading genetic diseases associated with infant mortality (Wirth et al., 2020; Singh et al., 2021). *SMN2*, a nearly identical copy of *SMN1*, cannot compensate for the loss of *SMN1* due to predominant skipping of exon 7 (Lefebvre et al., 1995; Cho and Dreyfuss 2010). Functions of *SMN2* remain unknown, although high demand of SMN in testis is partially met through an adult-specific switch in *SMN2* exon 7 splicing (Ottesen et al., 2016). While not intensively investigated, low levels of SMN also affects male fertility in adults suffering from mild SMA (Ottesen, Howell, Singh, Seo, Whitley and Singh 2016; Lipnick et al., 2019). Two currently approved therapies of SMA are based on the restoration of *SMN2* exon 7 inclusion (Singh et al., 2020b). Due to the fact that SMA is predominantly linked to defects in *SMN1* and that *SMN2*, a nearly identical copy of *SMN1*, is “available” in SMA patients, regulation of exon 7 splicing has been intensively investigated. Several cis-elements and transacting factors have been implicated in the regulation of *SMN* exon 7 splicing (Singh and Singh 2018) (Figure 1).

Splicing regulation is a complex process requiring precise definition of the splice sites (Shenasa and Hertel 2019). Recognition of the 5′ss by U1 snRNP is one of the earliest steps of assembly of the spliceosome that catalyzes the splicing reaction (Charenton et al., 2019). Once recruited, U1 snRNP can also define the upstream 3′ss through cross-exon interactions (De Conti et al., 2013). Similarly, U2 snRNP recruited at the 3′ss promotes recruitment of U1 snRNP at the downstream 5′ss through cross-exon interaction (De Conti et al., 2013). A critical C-to-T mutation at the 6^th^ exonic position of exon 7 (C6U substitution in RNA) was found to be the primary cause of *SMN2* exon 7 skipping (Lorson et al., 1999; Monani et al., 1999). Being close to the 3′ss, the C6U was assumed to weaken the 3′ss of the exon (Lim and Hertel 2001). Initially, it was proposed that C6U abrogates an enhancer associated with ASF/SF2 (Cartegni and Krainer 2002) (Figure 1). However, this claim was promptly challenged and a competing hypothesis suggesting that C6U creates a silencer associated with hnRNP A1 was put forward (Kashima and Manley 2003) (Figure 1). Our earlier work showed that C6U strengthens an extended inhibitory context (Exinct) at the 3′ss of exon 7 (Singh et al., 2004a; Singh et al., 2004c) (Figure 1). Additional factors that interact with C6U or in its vicinity were subsequently identified (Pedrotti et al., 2010; Singh and Singh 2018) (Figure 1).

The inhibitory nature of C6U was independently validated by *in vivo* selection in which relative significance of all 54 positions of *SMN* exon 7 was probed simultaneously (Singh et al., 2004b). Results of *in vivo* selection of exon 7 confirmed the presence of “Exinct” in the beginning of the exon 7 and revealed two additional regulatory regions termed as the “Conserved tract” and “3′-Cluster”. Located in the middle of exon 7, the “Conserved tract” exerts a positive effect on exon 7 splicing. The “3′-Cluster” is located towards the end of exon 7 and exerts a negative effect on exon 7 splicing (Figure 1). The most surprising finding of *in vivo* selection was the suboptimal nature of the 5′ss of exon 7 (Singh et al., 2004b; Singh 2007). In humans, the last exonic position in most cases is represented by a G residue. This G residue base pairs with a C residue of U1 snRNA (Lund and Kjems 2002). In addition, during catalytic core formation this G residue forms a base pair with a C residue of U5 snRNP (Lund and Kjems 2002). *In vivo* selection of the entire exon 7 revealed that an A residue at the last position of exon 7 constitutes the most inhibitory nucleotide that contributes to exon 7 skipping. Consistently, an A-to-G mutation at the last position of exon 7 (A54G substitution) fully restored *SMN2* exon 7 inclusion. A GA-rich enhancer in the middle of exon 7 has been found to be critical for *SMN2* exon 7 inclusion (Hofmann et al., 2000; Hofmann and Wirth 2002; Young et al., 2002). The enhancer constitutes a binding site for Tra2β1 and its associated factors (Figure 1). Of note, the effect of the A54G substitution was so strong that it promoted *SMN2* exon 7 inclusion even in the absence of this GA-rich enhancer (Singh et al., 2004b). A subsequent study confirmed that A54G destabilizes a terminal stem-loop structure (TSL2) and helps recruit U1 snRNP through extended based pairing with U1 snRNA at the 5′ss of exon 7 (Singh et al., 2007). It should be noted that risdiplam also interacts with A54 and helps recruit U1 snRNP (Campagne et al., 2019). It is worth mentioning that recruitment of engineered U1 snRNAs to sequences located downstream of the 5′ss of exon seven can also promote exon 7 inclusion (Singh et al., 2017a; Singh RN. and Singh NN. 2019).

Several studies have focused on the role of regulatory elements within *SMN* intron 7, the last intron of *SMN* genes (Singh and Singh 2018) (Figure 1). The discovery of the 15-nucleotide long intronic splicing silencer N1 (ISS-N1) spanning the region from the 10^th^ to 24^th^ positions of intron 7 revealed (for the first time) the strong inhibitory impact of the intronic element on *SMN2* exon 7 splicing (Figure 1). Deletion or an ASO-directed blocking of ISS-N1 fully restored *SMN2* exon 7 inclusion (Singh et al., 2006). Subsequent studies confirmed that among many potential targets for an ASO-based therapy for SMA, ISS-N1 was the leading contender (Hua et al., 2008). Upon successful completion of clinical trials, the ISS-N1-targeting ASO nusinersen (commercial name: Spinraza) was approved as the first drug for the treatment of SMA (Singh et al., 2017b; Bennett et al., 2019). ISS-N1 is a complex regulatory element that harbors two putative sites contacted by a single hnRNP A1 molecule (Beusch et al., 2017). The first five nucleotides of ISS-N1 also overlaps with an upstream 8-nucleotide-long GC-rich motif, sequestration of which by a short ASO promoted *SMN2* exon 7 inclusion and provided therapeutic benefits in mouse models of SMA (Singh et al., 2009; Keil et al., 2014). Another splicing silencer element located in intron 7 is created by a *SMN2*-specific A-to-G substitution at the 100^th^ position of intron 7 (Kashima et al., 2007). Intron 7 also contains two positive regulatory elements downstream of ISS-N1 (Miyaso et al., 2003; Singh et al., 2011). One of these elements is a binding site for TIA1 that is known to stimulate recruitment of U1 snRNP at the 5′ss (Singh et al., 2011). We have demonstrated that TIA1 is indeed a modifier of SMA in a gender-specific manner (Howell et al., 2017a). Here, we review the structural context of *SMN* exon 7 and its flanking introns 6 and 7. We focus on both probed and predicted secondary structures that have been implicated in regulation of *SMN* exon 7 splicing. We also discuss the structural context of mutations that profoundly impact *SMN* exon 7 splicing.

### Structure of *SMN* Exon 7

**FIGURE 2:**
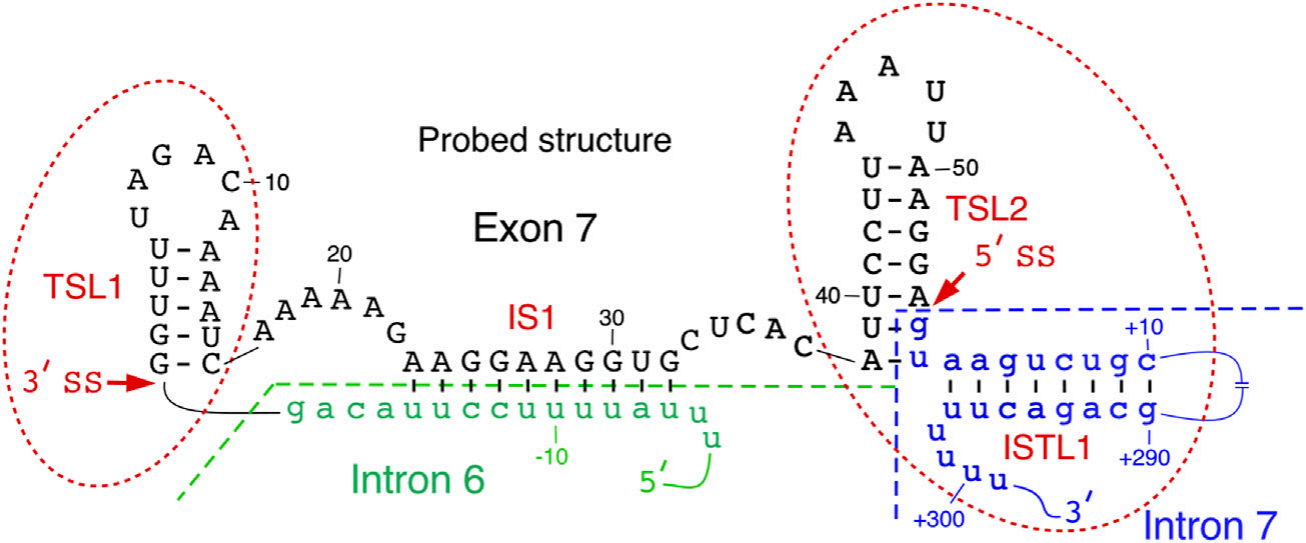
Local structure of *SMN* exon 7 and adjacent upstream/downstream intronic sequences. Existence of TSL2 and its effect on exon 7 splicing was also confirmed by mutational analysis. Intron 6 and intron 7 sequences are shown in small-case green and blue letters, respectively. Exon 7 sequence is shown in upper-case black letters. Numbering of nucleotides, neutral, positive and negative, starts from the first position in exon 7, first position of intron 7 and the last position in intron 6, respectively. The splice sites of exon 7 are indicated by the arrows. IS1 structure is boxed. Abbreviation: IL, internal loop; IS, internal stem; TSL, terminal stem loop.

Secondary structure of *SMN* exon 7 and the downstream intron 7 probed by enzymatic and chemical methods reveals the presence of two terminal stem-loop structures, TSL1 and TSL2 (Singh et al., 2004a; Singh et al. 2007; Singh et al. 2013) (Figure 2). Located at the 5′-end of exon 7, TSL1 sequesters several presumed cis-elements that define the 3′ss of exon 7. *SMN2* specific C6U is predicted to stabilize TSL1 by increasing the size of the stem in TSL1. However, results of enzymatic structure probing showed that the U residue at the 6^th^ exonic position is highly accessible. Hence, the six-nucleotide loop of TSL1 encompasses the UAGAC motif that may serve as a binding site of hnRNP A1, a negative regulator of *SMN2* exon 7 splicing. A recent study in the context of telomerase RNA has shown that hnRNP A1 preferentially interacts with its motif presented in the loop (Liu et al., 2017). The findings of this study are instructive as they highlight the viewpoint that a structural context plays a pivotal role in deciding the “fate” of RNA-protein interactions in a living cell, where limited protein supply requires binding of a given protein to its best RNA target. Supporting the inhibitory nature of TSL1, mutations predicted to abrogate this secondary structure stimulated *SMN2* exon 7 inclusion (Singh et al., 2004a). However, results of mutations should be interpreted with caution as the stimulatory effect of a given mutation on splicing could be due to accidental creation of an enhancer element and/or abrogation of a silencer element.

TSL2 is one of the most scrutinized structures of exon 7. TSL2 is formed by sequences at the 3′-end of exon 7. Compared to TSL1, the structure of TSL2 is more rigid due to its longer stem. Importantly, TSL2 sequesters the first two intronic positions that define the 5′ss of exon 7 (Figure 2). In principle, U1 snRNA has a potential to form an 11-bp long RNA:RNA duplex with the 5′ss of an exon formed between U1 snRNA and the last three exonic and the first eight intronic positions (Lund and Kjems 2002). Engineered U1 snRNAs capable of forming a “perfect” 11-nucleotide-long duplex with the 5′ss of exon 7 or downstream sequences have been shown to promote *SMN2* exon 7 inclusion (Singh et al., 2007; Singh et al., 2017a). In the case of *SMN* exon 7, U1 snRNP is predicted to form only 6 base pairs with the 5′ss of exon 7, deeming it suboptimal. Partial sequestration of the 5′ss of exon 7 by TSL2 imparts a strong inhibitory effect on splicing of *SMN2* exon 7. Consistently, mutations that disrupted TSL2 promoted *SMN2* exon 7 inclusion (Singh et al., 2007). Hence, a strong stimulatory effect of A54G substitution revealed by *in vivo* selection of the entire exon 7 could be attributed, at least in part, to the disruption of TSL2 (Singh 2007). Confirming the inhibitory role of TSL2, compensatory mutations that reinstated TSL2 restored skipping of *SMN2* exon 7 splicing. Interestingly, dinucleotide substitutions that strengthened TSL2 promoted exon 7 skipping, even in the context of *SMN1* (Singh et al., 2004a). These findings support the idea that transfactors interacting with splicing enhancers present within *SMN1* exon 7 are insufficient to promote exon 7 inclusion in the context of a rigid secondary structure sequestering the 5′ss of exon 7.

TSL1 and TSL2 are separated from each other by an internal stem, IS1, formed between sequences in the middle of exon 7 and the 3′-end of intron 6 (Figure 2). IS1 partially sequesters the polypyrimidine tract with a potential negative impact on the recognition of the 3′ss of exon 7 by U2 snRNP. IS1 also sequesters the “Conserved tract”, a positive regulator of exon 7 splicing (Singh et al., 2004b). Supporting the negative impact of IS1 on exon 7 splicing, substitutions at several positions within IS1 were selected for in our “*in vivo* selection of the entire exon 7” experiment (Singh et al., 2004b). In addition, stimulatory mutations in the 5′ strand of TSL1 stem are also predicted to disrupt IS1 by forming alternative structures. We hypothesize that an abrogated IS1 might make interactions between a positive transfactor(s) and the “Conserved tract” possible, which in turn will lead to destabilization of TSL2 and improved recruitment of U1 snRNP at the 5′ss of exon 7. IS1 may also serve as an “anchor” for an interaction(s) with splicing-modulating small molecules. For instance, risdiplam, the recently approved small molecule for SMA therapy has been shown to interact with an AG-rich motif located within IS1 (Sivaramakrishnan et al., 2017; Ratni et al., 2018; Singh NN. and Singh RN. 2019; Singh et al., 2020a).

### Structure of *SMN* Intron 7

**FIGURE 3:**
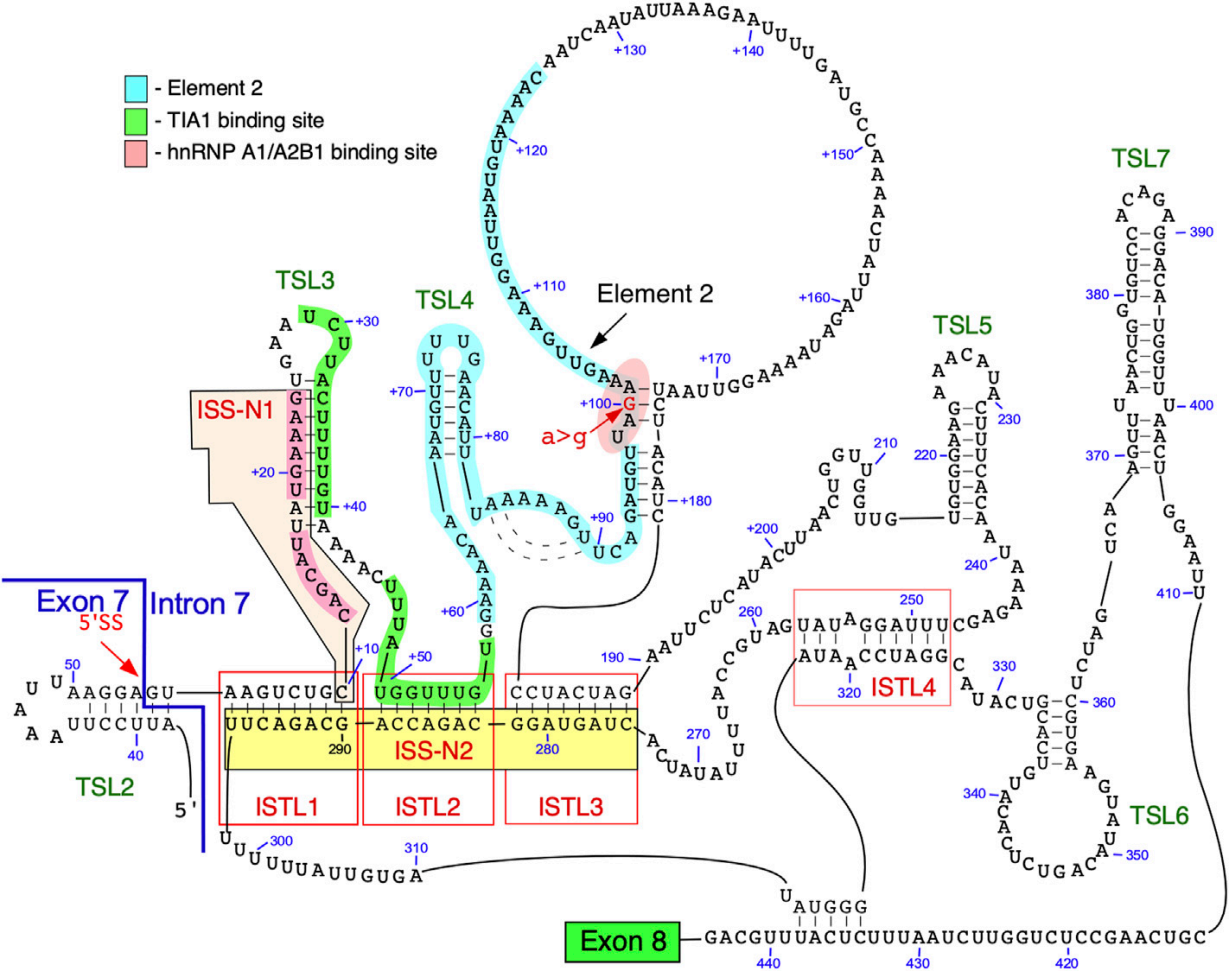
Secondary structure of *SMN* intron 7. The structure is based on combined probing by enzymatic and chemical methods. An exon 7/intron 7 junction as well as the 5′ss are indicated. Exon 8 is represented by a green box. Numbering of nucleotides, neutral and. positive, starts from the first position of exon 7 and the first position of intron 7, respectively. Binding sites of hnRNP A1/A2 and TIA1 are highlighted in pink and green, respectively. Element 2 sequence is highlighted in light blue. ISTL, internal stem formed by a long-distance interaction; TSL, terminal stem-loop; ISS-N2, intronic splicing silencer 2.

The secondary structure of intron 7 of *SMN2* probed using SHAPE revealed five terminal stem loops (TSL3, TSL4, TSL5, TSL6 and TSL7) and three internal stems formed by long-distance interactions (ISTL1, ISTL2 and ISTL3) (Singh et al., 2013; Singh et al., 2015b) (Figure 3). Among structures formed by intron 7, ISTL1 has been intensively investigated. ISTL1 is an 8-bp long duplex that sequesters the remaining portion of the 5′ss of exon 7. The cytosine residue at the 10^th^ intronic position (^10^C) is the last nucleotide of the 5′-strand of ISTL1. ^10^C also happens to be located at the first position of the 15-nucleotide-long ISS-N1 (Figure 3). The functional significance of ISTL1 was uncovered due to an unexpected finding that two 14-nucleotide ASOs, F14 and L14, produced an opposite effect on *SMN2* exon 7 splicing (Singh et al., 2010). While F14 promoted *SMN2* exon 7 inclusion by sequestering the first 14 nucleotides of ISS-N1, L14 triggered *SMN2* exon 7 skipping by sequestering the last 14 nucleotides of ISS-N1. The annealing positions of F14 and L14 differed by a single nucleotide as F14 sequestered ^10^C and L14 did not. Subsequent experiments revealed that F14 and L14 destabilize and stabilize ISTL1, respectively (Singh et al., 2013). The finding that L14 triggers *SMN2* exon 7 skipping by stabilizing ISTL1 shows that an ASO has a potential to drastically alter the structural context outside its annealing positions.

Two strands of ISTL1 are separated from each other by 279 nucleotides. Supporting the inhibitory nature of ISTL1, mutations that disrupted ISTL1 promoted *SMN2* exon 7 inclusion (Singh et al., 2013). Compensatory mutations that reinstate the disrupted ISTL1 restored the inhibitory effect of ISTL1. ISTL2 and ISTL3 are additional structures formed by long-distance interactions, they share a continuous 3′-strand with ISTL1. The continuous sequence encompassing the 3′-strands of ISTL1, ISTL2 and ISTL3 was termed ISS-N2 (Figure 3) (Singh et al., 2013). ASOs blocking different regions of ISS-N2 stimulate *SMN2* exon 7 inclusion, supporting the inhibitory nature of ISTL1, ISTL2, and ISTL3 on *SMN2* exon 7 splicing. The stimulatory effect of an ISS-N2-targeting ASO was maximal when ASO sequestered the 3′-strand of ISTL1. An ISS-N2-targeting ASO also showed therapeutic benefit in a mouse model of SMA (Howell et al., 2017b). These results underscored that deep intronic sequences associated with RNA structure could be exploited for therapeutic purposes.

The structural context of intron 7 has significance for a better understanding of RNA-protein interactions and their role in *SMN2* exon 7 splicing (Singh et al., 2015b). For instance, TSL3 and ISTL2 sequester the binding sites of TIA1 that is known to stimulate *SMN2* exon 7 inclusion (Singh et al., 2011). One of the two putative binding motifs of hnRNP A1 present within ISS-N1 is located within a loop (Figure 3). Based on the structural context, we hypothesize that the strong binding site for hnRNP A1 presented in the loop of the stem-loop structure enables more efficient recruitment of this protein, which in turn renders the 5′ss of exon 7 inaccessible for the recruitment of U1 snRNP (Singh et al., 2015a). Furthermore, an ISS-N1 targeting ASO not only blocks the binding site of hnRNP A1 but also makes the TIA1 binding site “available” for interaction with TIA1 due to disruption of TSL3. Similarly, an ISS-N2-targeting ASO makes U1 snRNP and TIA1 binding sites accessible by disrupting ISTL1 and ISTL2, respectively.

**FIGURE 4:**
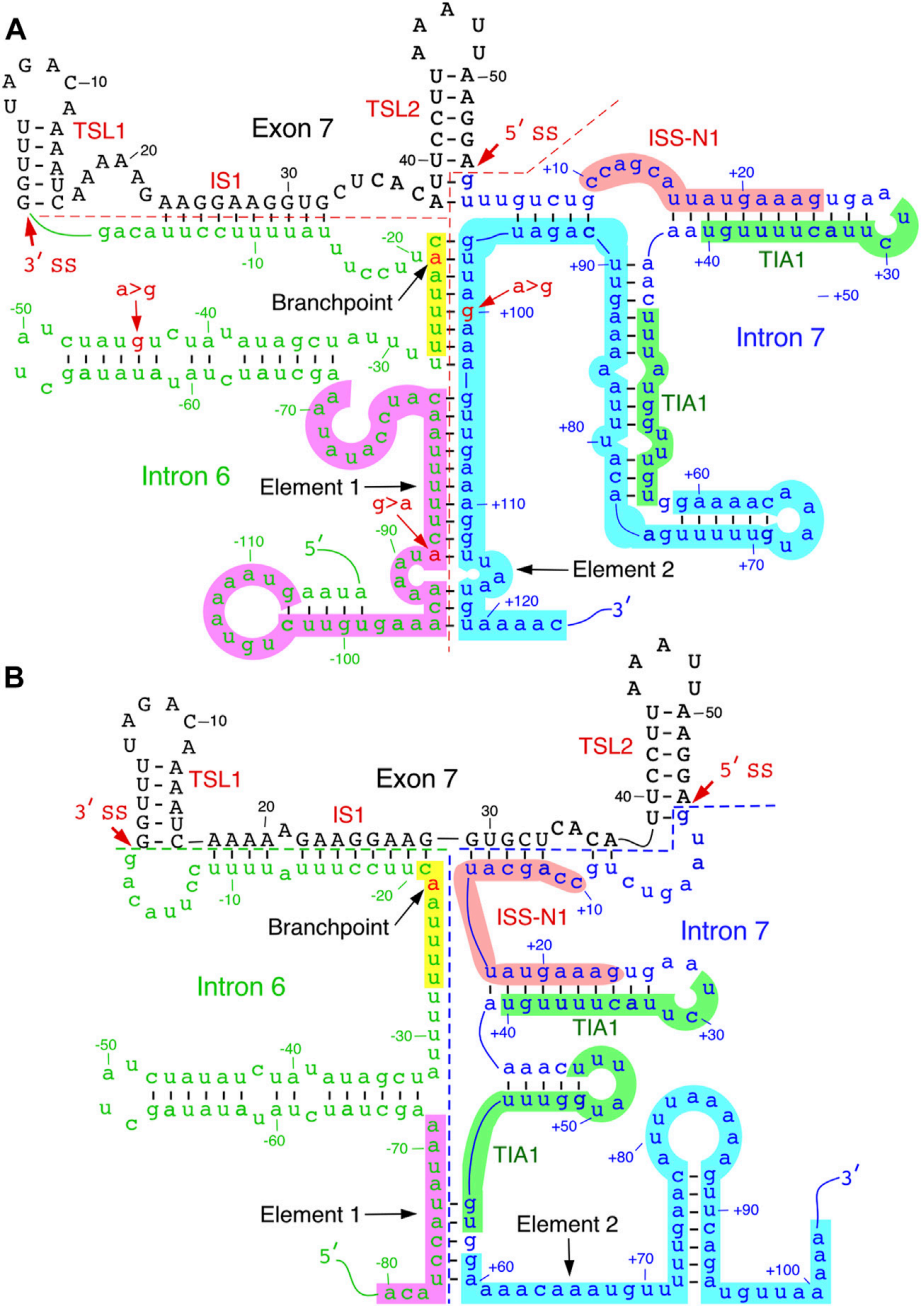
Predicted inter-intronic structures. **(A).** Secondary structure formed between elements 1 and 2 located within introns 6 and 7, respectively. The structure sequesters both TIA1 binding sites. Numbering of nucleotides, negative, neutral and negative, starts from the last position of intron 6, first position of exon 7 and the first position of intron 7, respectively. Element 1 is highlighted in purple; branch point sequence, in yellow, with “A” indicated in red. Other markings and abbreviations are same as shown in Figure 3. **(B).** An alternative secondary structure of element 2. The structural context changes the positioning of the TIA1 binding sites. Markings and abbreviation are the same as in panel A.

Element 2, a positive regulator of *SMN* exon 7 splicing, is located downstream of the TIA1 binding site within intron 7 (Figures 1, 3) (Miyajima et al., 2002; Miyaso et al., 2003). The probed structure of intron 7 places Element 2 in both the structured region and the loop (Figure 3). Interestingly, the *SMN2*-specific A-to-G mutation at the 100^th^ position of intron 7 falls within the stem region of Element 2 (Figure 3). Of note, A-to-G mutation at the 100^th^ intronic position has been suggested to create a binding site for hnRNP A1, a negative regulator of *SMN* exon 7 splicing (Kashima et al., 2007). At the same time, deletion of Element 2 (together with A100G) has been shown to have a negative effect on *SMN* exon 7 splicing (Miyaso et al., 2003). These contradictory findings could be explained by the presence of multiple overlapping cis-elements within Element 2, a 66 nucleotide-long sequence (Miyaso et al., 2003). The strongest stimulatory effect associated with the region within Element 2 corresponds to TSL4 that harbors a U-rich loop (Figure 3). An alternative structure in this region is predicted to form a different stem-loop structure with an A-rich sequence within the loop (Figure 4) (Miyaso et al., 2003). However, the significance of any of these structures have not yet been investigated. Of note, transfactor(s) that interact with Element 2 remain unknown.

### Inter-Intronic Structures

Folding algorithms, including mfold, RNAfold and ScanFold, can predict secondary structures with high confidence (Wang et al., 2008; Rouse et al., 2022); notably, the latter program provides metrics and models describing base pairs with likely functionality (Andrews et al., 2018). Secondary structures of *SMN* exon 7 and downstream intron 7 predicted by the above algorithms have generally agreed with the probed structures. Considering the structure of *SMN* intron 6 has not yet been probed, one can gain valuable insights from the predicted secondary structure. *SMN* intron 6 harbors fourteen copies of Alu-like elements, some of them are present as inverted repeats (Ottesen et al., 2017). One of these Alu elements is used as an exon, although it is predominantly skipped or/and degraded via nonsense-mediated decay (Seo et al., 2016). Secondary structures formed by inverted Alu repeats within intron 6 have been implicated in the generation of *SMN* circRNAs (Ottesen et al., 2019; Ottesen and Singh 2020). At the same time, a large deletion encompassing all Alu-elements of intron 6 was found to have no impact on splicing of *SMN* exon 7 (Singh et al., 2004a). However, sequence motifs close to the 3′-end of intron 6 have been shown to modulate *SMN* exon 7 splicing (Singh and Singh 2018). One such motif is Element 1 that imparts a negative impact on *SMN* exon 7 (Miyajima et al., 2002). Deletion or an ASO-mediated sequestration of Element 1 has been shown to promote *SMN2* exon 7 inclusion (Miyajima et al., 2002; Osman et al., 2016). One of the predicted structures of exon 7 and its flanking intronic sequences places the middle of Element 1 in an internal stem formed with sequences of intron 7 (Figure 4A). Interestingly, the 3′-strand of this internal stem comes from a portion of Element 2. Hence, it is likely that this inter-intronic RNA:RNA duplex suppresses the positive effect of Element 2 on exon 7 inclusion.

Formation of an RNA:RNA duplex between two neighboring introns leads to looping out of an exon which might trigger exon skipping. In addition to the inter-intronic structure that involves sequences of Elements 1 and 2, other predicted structures reveal additional RNA:RNA duplexes formed between introns 6 and 7. One such structure is created by base pairing between a sequence located downstream of Element 1 and the sequence located in the middle of Element 2. Factors such as hnRNP A1/A2 have been implicated in exon skipping through a looping out mechanism (Martinez-Contreras et al., 2006). Considering inter-intronic interactions/structures have potential to bring different hnRNP A1/A2 binding sites of in close proximity, it is likely that skipping of *SMN2* exon 7 is facilitated, at least in part, by a looping-out mechanism aided by inter-intronic RNA structures. An additional hypothesis would be that the inter-intronic RNA:RNA duplexes enforce the neighboring intra-intronic structures that sequester the positive regulatory elements in the vicinity of the splice sites of exon 7. The “looping out” hypothesis and “sequestration of splice sites due to RNA structure” hypothesis do not have to be mutually exclusive. The last fifty nucleotides of intron 6 harbor critical splicing regulatory elements, including the polypyrimidine tract and branchpoint. The predicted secondary structure in this region places most of the polypyrimidine tract in the internal stems. The precise location of *SMN* intron 6 branchpoint has not been identified yet. Nonetheless, the UUUUAAC motif corresponding to the consensus mammalian branchpoint motif YNYURAY is locked in a predicted RNA:RNA duplex (Figure 4A) (Gao et al., 2008). In an alternative predicted structure, the UUUUAAC motif is placed in the loop region, while positioning of ISS-N1 and the TIA1-binding sites is drastically changed (Gao et al., 2022) (Figure 4B). In this alternative structure, the first half of ISS-N1 interacts with exon 7 and blocks a portion of the “Conserved tract”, the positive regulator of exon 7 inclusion (Figure 4B). Mutations within the 5′ half of ISS-N1 are known to promote exon 7 inclusion (Supplementary Figure S1). Future experiments will reveal if the stimulatory effect of these mutations is linked, at least in part, to the RNA structure.

### Assessing Propensity for Secondary Structure Formation

**FIGURE 5:**
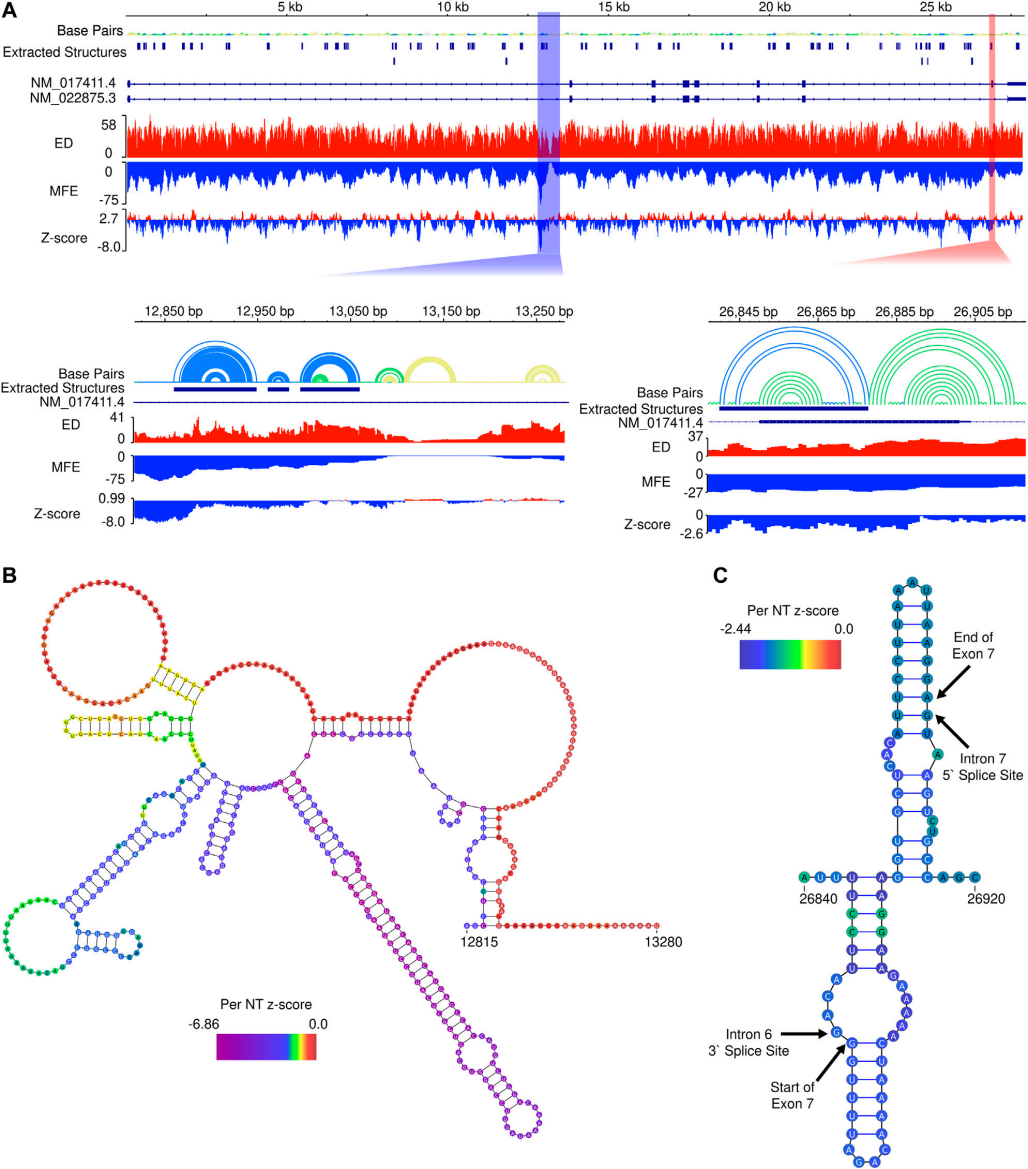
*In silico* ScanFold results for the pre-mRNA of *SMN2*. **(A).** At the top, an IGV representation of the whole *SMN2* pre-mRNA transcript with 6 data tracks: a base pair (arc diagram) track, a track showing ScanFold extracted structures where base pairs with z-score < -2, -1 and 0 are indicated in blue, green and yellow, respectively; a track of transcripts (with introns as lines and exons as boxes), an ensemble diversity (ED) track, a minimum free energy (MFE) track, and a ΔG z-score track. Below the whole transcript are two zoomed in regions. Highlighted by the blue box is a region of *SMN2* with the lowest z-score and both the lowest and highest MFE regions in the transcript. Highlighted by the red box is Exon 7, which contains one of the two extracted structures present in an exonic region. **(B).** The ScanFold informed 2D model of the blue highlighted region from panel A is shown with the per nucleotide (NT) z-score overlaid on the model. **(C).** The ScanFold informed 2D model of the red highlighted region from panel A is shown with the per nucleotide (NT) z-score overlaid on the model. Here, the boundaries of intron splice sites and the start and stop site of exon 7 are labelled.

To assess potential for additional functional RNA structural elements, we performed preliminary scans of the pre-mRNA sequence of *SMN2* using the ScanFold tool (Andrews et al., 2018) (Figure 5A). Within *SMN2*, ScanFold identified 84 significantly stable structures (containing base pairs with <-2 average z-score) suggesting these structures have a functional role to play (e.g., the *SMN2* intronic structure in Figure 5B). Only two of these 84 highlighted structures overlap exonic sequence, while the rest resides in introns. Notably, exon 7 is spanned by one of the significantly stable ScanFold predicted exonic structures (Figure 5C) and the results recapitulate portions of the two previously determined hairpin structures TSL1 and TSL2 (Singh et al., 2007; Singh et al., 2013).

Interestingly, the region of *SMN2* which had the lowest ΔG z-score also had the lowest (most negative) minimum free energy (MFE), which is not always the case, as some relatively unstable (ΔG) regions may have *ordered* stability bias. Additionally, this region is adjacent to the region of highest (least stable) MFE predictions in *SMN2* (Figure 5B). Notably, the region downstream of this identified structural motif also had the lowest ensemble diversity (ED). In this case, the lack of conformational diversity indicated by low ED suggests that the nucleotides here are likely to be single-stranded in most or all conformations, possibly to facilitate intermolecular interactions with regulatory transfactors.

We also employed the ScanFold program to examine the local structural context of the 5-nucleotide motifs occupying the 11^th^ to 15^th^ positions of *SMN2* intron 7. This region falls within the ISS-N1 sequence and has recently been intensively investigated (Gao et al., 2022). One of the putative hnRNP A1 motifs, CAGCA, happens to occupy the 11^th^ to 15^th^ positions of *SMN2* intron 7 (Hua et al., 2008). Local structure predicted by ScanFold places the last four residues of the inhibitory CAGCA motif within a stem (Supplementary Figure S1). Six mutants that promoted *SMN2* exon 7 inclusion by abrogating the CAGCA motif bring noticeable changes in the local structural context (Gao et al., 2022) (Supplementary Figure S1). The stimulatory effect of these mutations could be due to both, abrogation of the negative hnRNP A1 motif and creation of a novel positive motif with the enhanced accessibility in the newly formed loop. We also observed changes in the local structural context in case of other mutants that maintained the inhibitory context while abrogating the putative motif associated with hnRNP A1 (Supplementary Figure S1). Interestingly, a single U-to-C substitution (from UUUUU to UUUCU) transformed a stimulatory motif into an inhibitory motif (Gao et al., 2022). This change retains the positioning of these 5-nucleotide motifs within the loop (Supplementary Figure S1). These results suggest that both positive and negative regulatory transfactors preferentially interact with their cognate binding sites in the single-stranded region. Future studies will reveal if additional features of high-order structure provide secondary contacts for RNA-protein interactions. Of note, the role of secondary interactions for an enhanced affinity has been reported for a factor known to promote self-splicing of a group II intron (Wank et al., 1999; Singh et al., 2002).

The ScanFold program identified 84 regions with significant thermodynamic stability throughout the pre-mRNA of *SMN2*. These regions have an ordered sequence arrangement, which displays higher than expected stability compared to randomized sequences of identical nucleotide composition. *SMN2* contains 42 Alu elements located within its intronic sequences that represent ∼39% of the *SMN2* gene (Ottesen et al., 2017). The huge repertoire of circRNAs produced by *SMN* genes is attributed to the RNA duplexes formed between inverted Alu repeats (Ottesen et al., 2019). Of the 84 significantly stable regions identified by ScanFold, 54 overlapped Alu elements, including 47 regions that are fully contained within an Alu element. Of the 42 Alu elements located within *SMN2* pre-mRNA, 30 overlapped regions of significant stability. These regions of ordered sequence and enhanced stability are prime candidates for future investigations in structure-function mechanisms associated with Alu elements present in *SMN2*. Overall, these preliminary results strongly indicate that RNA secondary structure may be playing functional roles, most significantly in processing pre-mRNA into a mature transcript(s).

## Concluding Remarks

The role of RNA structure in pre-mRNA splicing is a topic of growing significance. Local RNA structures are formed instantaneously as soon as a transcript emerges from the RNA polymerase. Initial RNA structures transition to more favorable structures formed by long-range interactions. However, the assessment of structural transitions in the cell remains a challenging task: for example, RNA helicases break certain secondary structures with high specificity. Protein factors tightly interacting with RNA may also impede the transition from one RNA structure into another. Potentially hundreds of proteins could be recruited during the removal of a single intron. Some of these RNA-protein interactions are expected to be non-specific due to the propensity for negatively charged RNA molecules to form electrostatic interactions with positively charged (basic) residues presented by proteins. In contrast, other RNA-protein interactions rely on certain RNA motifs. RNA structures may provide specificity by placing a given motif into a unique context. A previous report uncovered the structural context of several splicing factors that were initially thought to have preference for small linear motifs (Dominguez et al., 2018). Findings summarized in this study represent the tip of the iceberg as methods to uncover genome-wide RNA-protein interactions in the cell are still at their infancy.

Two steps of transesterification involved in the removal of every intron during pre-mRNA splicing are RNA catalyzed reactions resembling those of the self-splicing group II introns (Smathers and Robart 2019). However, unlike in self-splicing group II introns where an intronic structure alone brings and holds the splice sites together, the role of intronic sequences in pre-mRNA splicing has been assigned to recruiting snRNPs that bring and hold the splice sites together. Owing to the diverse nature of intronic sequences, the mechanism of snRNPs recruitment differs from one intron to another. Currently, there is no explanation why certain introns are removed more efficiently than others despite the comparable strength of their splice sites. The answer to this question may partly lie in the RNA structures that may sequester the splice sites and/or fold in a manner that brings the splice sites of an intron in close proximity. The generation of circRNAs provides the most convincing example of how RNA structures bring a downstream 5′ss to an upstream 3′ss for backsplicing. The process of circRNA generation competes with the forward splicing that produces the linear transcripts.

Aberrant splicing is associated with many genetic disorders. SMA happens to be one of the model diseases in which modulation of splicing employing ASOs and small molecules have conferred therapeutic benefits. The therapeutic compounds utilized in treating SMA were selected based on their ability to restore *SMN2* exon 7 inclusion. Several cis-elements and transacting factors have roles implicated in regulation of *SMN* exon 7 splicing. Probed RNA structures place the 5′ss of *SMN* exon 7 in a highly sequestered structural context encompassing TSL2 and ISTL1. TSL2 and ISTL1 represent examples of an inhibitory local stem-loop structure and an inhibitory structure formed by long-distance interactions, respectively. The formation of TSL2 and ISTL1 is proposed to provide a platform for the recruitment of negative regulatory factors, including hnRNP A1/A2. Another inhibitory structure, TSL1, formed at the 3′ss of *SMN2* exon 7 shifts the hnRNP A1/A2 binding site into the loop. An additional hnRNP A1/A2 binding site is located in the partially structured region of *SMN2* intron 7. Overall structural context of *SMN2* exon 7 and its flanking intronic sequences renders binding sites inaccessible for stimulatory transfactors that recruit U1 and U2 snRNPs at the 5 and 3′ ss of exon 7, respectively. Abrogation of a long-distance interaction similar to that of ISTL1 has been associated with the X-linked leukodystrophy Pelizaeus–Merzbacher disease (Taube et al., 2014). There is a growing interest to predict such structures as they may offer novel therapeutic targets for a number of diseases (Bernat and Disney 2015; Pervouchine 2018). Although beyond the scope of this review, recent studies on the short- and long-range RNA-RNA interactome provide interesting insights into replication of viruses, including SARS-CoV-2 (Smyth et al., 2018; Ziv et al., 2020). Future studies will reveal if these structures could be exploited for therapeutic purposes.

The roles of inter-intronic structures are the least appreciated aspects of *SMN* exon 7 splicing. Folding algorithms support formation of structures between introns 6 and 7 with potential to prime exon 7 to skipping if these structures are not immediately disrupted. One mechanism to avoid inter-intronic interactions is through fast removal of either intron 6 or intron 7 before structures are formed. The order in which *SMN* introns are removed remain largely unknown. In general, large introns are removed later than the smaller introns (Kim et al., 2017). Considering *SMN* intron 6 is > 13-fold larger than intron 7, its removal is expected to happen after the removal of intron 7. It is possible that a delayed removal of intron 7 further delays the removal of intron 6 leading to skipping of exon 7. We hypothesize that ASOs and small molecules that promote *SMN2* exon 7 inclusion preferentially stimulate a fast removal of intron 7 through strengthening of the 5′ss of exon 7. Future studies will determine if the disruption of inter-intronic structures present therapeutic avenues for the treatment of SMA.

Available tools of RNA structure prediction and methods of RNA structure probing have provided important insights into our understanding of *SMN* exon 7 splicing. Yet, much remains to be learned about how RNA structures decide the fate of RNA-protein interactions in the context of *SMN* exon 7 splicing. Several mutations in the intron 7 have been recently shown to restore *SMN2* exon 7. It will be interesting to see if RNA structure provides a mechanistic basis to explain the consequences of these mutations. Forward splicing affects backsplicing and introns used for backsplicing are generally spliced at the end (Kim et al., 2017). Considering that the 3′ss of exon 6 is used for backsplicing (Ottesen et al., 2019), it will be important to know how backsplicing-associated inter-intronic structures between intron 5 and downstream introns impact the forward splicing events, including removal of introns 6 and 7. Splicing is coupled to transcription elongation as many factors are recruited to the nascent pre-mRNA by RNA polymerase II before the termination of transcription (Saldi et al., 2016). The structure of a nascent RNA affects transcription elongation and vice-versa (Saldi et al., 2016). Hence, compounds that promote *SMN* exon 7 splicing through RNA structures formed during transcription elongation may provide yet another avenue for SMA therapy. Despite tremendous progress, currently approved therapies do not fully meet the needs of SMA patients (Singh 2019). A proper understanding of an RNA structure of *SMN* pre-mRNA will make a profound contribution to our understanding of splicing regulation of *SMN* genes. Novel findings emerging from the studies pertaining to the structure of *SMN* pre-mRNA will also shape the future therapeutic development of SMA and other diseases amenable by splicing modulation.
