# Supplementary material for: Structural Context of a Critical Exon of Spinal Muscular Atrophy Gene
Source: Front Mol Biosci. 2022 Jul 1;9:928581. doi: 10.3389/fmolb.2022.928581 (PMC9283826; doi:10.3389/fmolb.2022.928581)

Supplementary Figure S1. RNAfold-generated secondary structures of *SMN2* Exon 7 with its flanking portions of intron 6 and intron 7. All models are composed of the same input sequence, except for the 5-mer motif which contains stimulatory and inhibitory sequences as reported in (Gao et al. 2022). All models have the first three nucleotides of exon 7 highlighted in green (model positions 79-81), the last three nucleotides of exon 7 highlighted in red (model positions 130-132), and the intron 7, 5-mer motif (which influences splicing) is highlighted in blue (model positions 143-147). **(A)**. The wild type (WT) 2D model. **(B).** A 2D model with stimulatory sequence AACUC. **(C).** A 2D model with stimulatory sequence CCCAC. **(D).** A 2D model with stimulatory sequence CCCUC. **(E).** A 2D model with stimulatory sequence UUUUU. **(F)**. A 2D model with stimulatory sequence CCCGC. **(G)**. A 2D model with stimulatory sequence AUGCA. **(H)**. A 2D model with inhibitory sequence UUUCU. **(I).** A 2D model with inhibitory sequence AGGCA. **(J).** A 2D model with inhibitory sequence UCCUC. **(K)**. A 2D model with inhibitory sequence ACCGC. **(L).** A 2D model with inhibitory sequence CCAAC. **(M).** A 2D model with inhibitory sequence ACCUC.

Reference:

Gao, Y., Lin, K.T., Jiang, T., Yang, Y., Rahman, M.A., Gong, S., Bai, J., Wang, L., Sun, J., Sheng, L. *et al.* (2022) Systematic characterization of short intronic splicing-regulatory elements in SMN2 pre-mRNA. *Nucleic Acids Res*, **50**, 731-749.

Supplementary Figure S1.

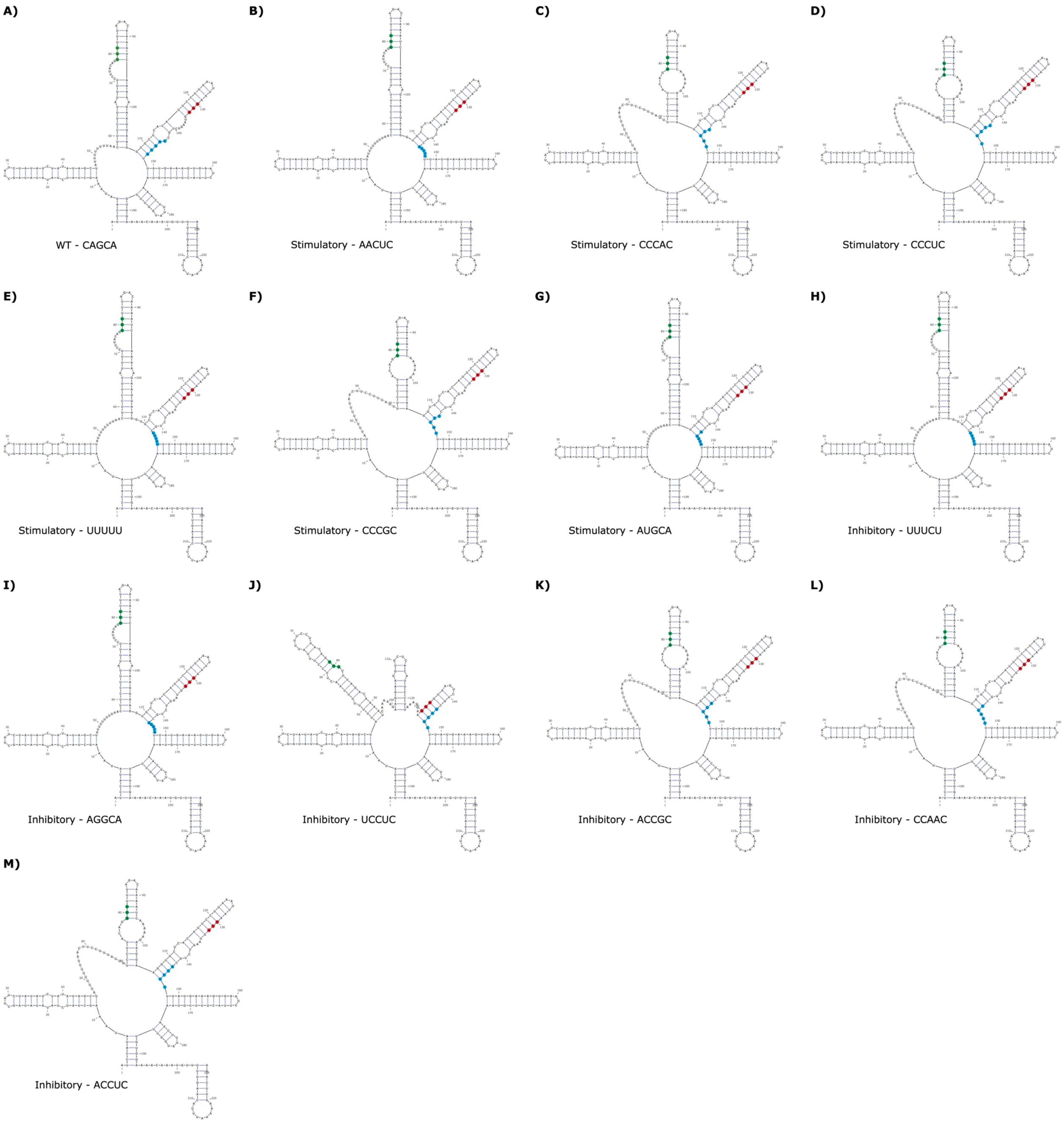

Supplement: Supplementary file 1 [file DataSheet1.PDF]
